# Oscillatory dynamics of Gestalt perception in schizophrenia revisited

**DOI:** 10.3389/fpsyg.2014.00068

**Published:** 2014-02-04

**Authors:** Kevin M. Spencer, Shahab Ghorashi

**Affiliations:** ^1^Research Service, Veterans Affairs Boston Healthcare SystemBoston, MA, USA; ^2^Department of Psychiatry, Harvard Medical SchoolBoston, MA, USA

**Keywords:** schizophrenia, visual perception, Gestalt, gamma oscillation, event-related potential

## Abstract

**Background:** Abnormalities in **γ** oscillations (30–100 Hz) in the scalp-recorded electroencephalogram (EEG) have been proposed to reflect neural circuitry abnormalities in schizophrenia. Oscillations in the γ band are thought to play an important role in visual perception, mediating the binding of visual features into coherent objects. However, there is relatively little evidence to date of deficits in γ-mediated processes associated with Gestalt perception in schizophrenia.

**Methods:** Fourteen healthy control subjects (HC) and 17 chronic schizophrenia patients (SZ) discriminated between illusory Kanisza Squares and No-Square control stimuli, indicating their judgment with a manual button press. Time-frequency decomposition of the EEG was computed with the Morlet wavelet transform. Time-frequency maps of phase locking factor (PLF) values were calculated for stimulus- and response-locked oscillations.

**Results:** HC and SZ did not differ in reaction time, error rate, an early ERP effect associated with Gestalt processing, nor an early visual-evoked γ oscillation. Two response-locked high γ effects had greater PLF for Square than No-Square stimuli in HC, and the reverse pattern in SZ. One of these effects was correlated with thought disorder symptom ratings in SZ.

**Conclusions:** SZ demonstrated abnormalities in γ oscillations associated with the perception of Gestalt objects, while their early visual-evoked γ activity was mostly normal, contrary to previous results. This study supports the hypothesis that high-frequency oscillations are sensitive to aspects of psychosis.

## Introduction

Abnormalities in γ oscillations (30–100 Hz) in the scalp-recorded electroencephalogram (EEG) have been proposed to reflect cortical circuitry abnormalities in schizophrenia, particularly reduced inhibition of pyramidal cells by fast-spiking interneurons (Kwon et al., 1999; Gonzalez-Burgos and Lewis, 2008; Woo et al., 2010). Classically, γ oscillations have been thought to play an important role in visual perception, mediating the binding of visual features into coherent objects (Singer and Gray, 1995; Singer, 1999; but see Thiele and Stoner (2003), Palanca and DeAngelis (2005), Lima et al. (2010). Thus, it is perhaps surprising that while γ oscillation abnormalities have been reported in various domains in schizophrenia, such as auditory (e.g., Kwon et al., 1999; Leicht et al., 2010) and visual sensory function (e.g., Spencer et al., 2008a; Grützner et al., 2013), working memory (Cho et al., 2006; Haenschel et al., 2009), and motor control (Ford et al., 2008), to date relatively little evidence of γ abnormalities associated with visual object perception in schizophrenia has been reported. Rather, investigations of oscillatory activity during visual perception tasks involving feature binding have mainly reported abnormalities in β frequency oscillations (13–30 Hz), as we review below.

In the first study of γ oscillations related to visual feature-binding in schizophrenia, Spencer et al. (2003) found that the early visual-evoked γ oscillation (Vγ1; ~25–40 Hz, 80–120 ms) was evoked for Gestalt (Kanisza square) but not control stimuli in control subjects. In schizophrenia patients neither stimulus type evoked Vγ1. While this deficit was suggestive of an abnormality in an oscillatory feature-binding process, Vγ1 has been associated with attentional and memory processes (Herrmann et al., 2004), rather than feature binding *per se* (Tallon-Baudry et al., 1996). A follow-up study examining Vγ1 in a simple oddball task also found a deficit in schizophrenia patients, suggesting that the early perceptual process manifested by this oscillation was impaired in a task—and stimulus-independent manner (Spencer et al., 2008a). Further studies have reported deficits in visual-evoked γ oscillations in schizophrenia (Krishnan et al., 2005; Wynn et al., 2005; Grützner et al., 2013; Sun et al., 2013).

In a second study utilizing the same Gestalt perception task with Kanisza squares, Spencer et al. (2004) investigated oscillations that preceded and were phase-locked to the time of the manual response, reasoning that such response-locked oscillations might be more related to perceptual decision-making processes than stimulus-locked oscillations. The authors found a response-locked β oscillation (~24 Hz) in schizophrenia patients that was elicited when they detected the Gestalt stimulus, but not the control stimulus. A similar oscillation was found in the low γ band (~37 Hz) of the control subjects. The Gestalt effect (Square minus No-Square) on the patients' β oscillation was positively correlated with particular psychotic symptoms including thought disorder, disorganization, and visual hallucinations. The correlation between disorganization symptoms and the β Gestalt effect was consistent with the relationships observed between disorganization and Gestalt perception by Silverstein et al. (2000), Uhlhaas et al. (2005, 2006a). While Spencer et al. (2004) proposed that the β oscillation in patients was equivalent to the γ oscillation in controls but at a lower frequency, the sparse electrode array used in that study did not allow for a detailed comparison of the scalp topographies of these oscillations to establish whether or not these oscillations might have similar neural generators.

Uhlhaas and colleagues have published a series of studies examining the oscillatory correlates of Gestalt perception in schizophrenia using “Mooney” face stimuli (Mooney and Ferguson, 1951). Uhlhaas et al. (2006b) reported that induced γ oscillatory activity in the 40–70 Hz band did not differ between patients and controls, but inter-electrode phase synchrony in the β band evoked by “Mooney” faces was reduced in patients. Face-evoked β synchrony was positively correlated with positive symptoms, similar to the correlations between the β Gestalt effect and positive symptoms in Spencer et al. (2004). Reductions in γ band phase synchrony in patients were found in the face but also the no-face conditions. However, in a study employing the same task and using magnetoencephalography, Grützner et al. (2013) found that γ power in the response to the offset of upright compared to inverted face stimuli was reduced in schizophrenia patients. Most recently, the clearest example of a deficit in γ activity related to Gestalt perception in schizophrenia was reported by Sun et al. (2013), who found reduced Gestalt effect on a high γ induced oscillation in first-episode patients.

Thus, the evidence for γ oscillation deficits related to visual feature-binding in schizophrenia is rather sparse, despite the evidence for an important role of γ in visual feature-binding (Singer and Gray, 1995; Singer, 1999), and the accumulation of behavioral findings pointing to impaired visual integrative processes in this disorder (reviewed in Uhlhaas and Silverstein, 2005; Butler et al., 2008). In the present study we revisited this issue by replicating our Gestalt studies utilizing Kanisza stimuli. As in our previous studies (Spencer et al., 2003, 2004), subjects discriminated between Kanisza square and control stimuli. Here we used a dense electrode array to obtain more detailed information about the spatial topography of the oscillations under study. We also made a modification to the experimental paradigm by presenting the stimuli briefly, instead of remaining present until the manual response was issued, to test whether response-locked oscillations might be driven in part by the physical persistence of the stimuli. We predicted that γ abnormalities in schizophrenia patients would be correlated with positive symptom ratings, specifically hallucinations, thought disorder, and/or disorganization, as in our previous findings (Spencer et al., 2004).

## Materials and methods

### Subjects

This study was approved by the Institutional Review Boards of the Veterans Affairs Boston Healthcare System and Harvard Medical School. After a complete description of the study to the subjects, written informed consent was obtained. All subjects were paid for their participation.

Healthy control subjects (HC; *N* = 14, 2 females) and chronic schizophrenia patients (SZ; *N* = 17, 1 female) participated in the study. HC were recruited from the community. SZ were recruited from mental health services at the VA Boston Healthcare System, and were diagnosed according to DSM-IV criteria (First et al., 1995). All subjects were selected without regard for ethnicity, and met our standard inclusion/exclusion criteria: (1) age between 18 and 55 years; (2) right-handed as assessed by the Edinburgh handedness inventory (Oldfield, 1971) (so that possible hemispheric lateralization effects would not be obscured by left-handers with reduced or reversed functional laterality); (3) no history of electroconvulsive treatment; (4) no history of neurological illness, including epilepsy; (5) no history of alcohol or drug dependence, nor abuse within the last year, nor long duration (>1 year) of past abuse (DSM-IV criteria); (6) no present medication for medical disorders that would have deleterious EEG, neurological, or cognitive functioning consequences; (7) verbal IQ above 75; (8) no alcohol use in the 24 h prior to testing; and (9) English as a first language. In addition, HC were screened for the presence of an Axis-I disorder using the SCID-Non-Patient edition (First et al., 2002), and were also excluded if they reported having first-degree relative with an Axis I disorder.

Demographic and clinical data are presented in Table 1. Clinical symptoms were assessed using the Scale for the Assessment of Positive Symptoms (SAPS) (Andreasen, 1984) and the Scale for the Assessment of Negative Symptoms (SANS) (Andreasen, 1983). The final SZ and HC groups did not differ in sex proportions, age, or parental socio-economic status (PSES; Hollingshead, 1965). The diagnostic composition of the SZ group was 7 paranoid, 7 undifferentiated, and 3 schizoaffective. All of the patients were taking atypical antipsychotics at the time of the experiment. One patient was also receiving a typical antipsychotic. Antipsychotic medication dosages were converted to chlorpromazine equivalents (Stoll, 2001; Woods, 2003).

**Table 1:** **Demographic and clinical variables and between-group comparisons for the healthy control (HC) and schizophrenia patient (SZ) groups**.

	**HC (*N* = 14)**	**SZ (*N* = 17)**	**Statistic**	***p***
Age (years)	43.6 ± 6.5	43.8 ± 9.3	*t*_(29)_ = −0.041	0.967
Parental socio-economic status	2.6 ± 1.2	2.4 ± 0.8	*t*_(28)_ = 0.545	0.590
# trials per subject	1.8 ± 13	1.7 ± 17	*t*_(29)_ = 0.300	0.766
Age of onset (years)		24.2 ± 6.8		
Positive symptom total (SAPS)		9.9 ± 6.6		
Negative symptom total (SANS)		9.2 ± 3.3		
Medication dosage (chlorpromazine equivalents)		range 50–1200		

*Mean ± SD are given for each variable*.

### Stimuli and experimental design

Subjects were seated in a quiet, dimly-lit room, 1 m in front of a computer monitor upon which the visual stimuli were presented. Subjects were instructed to discriminate between Kanisza Square and control No-Square displays (90 trials per condition), pressing a button with one hand for square-present, and another button with the other hand for square-absent. The response hand assignment was counterbalanced across subjects.

**Figure 1:**
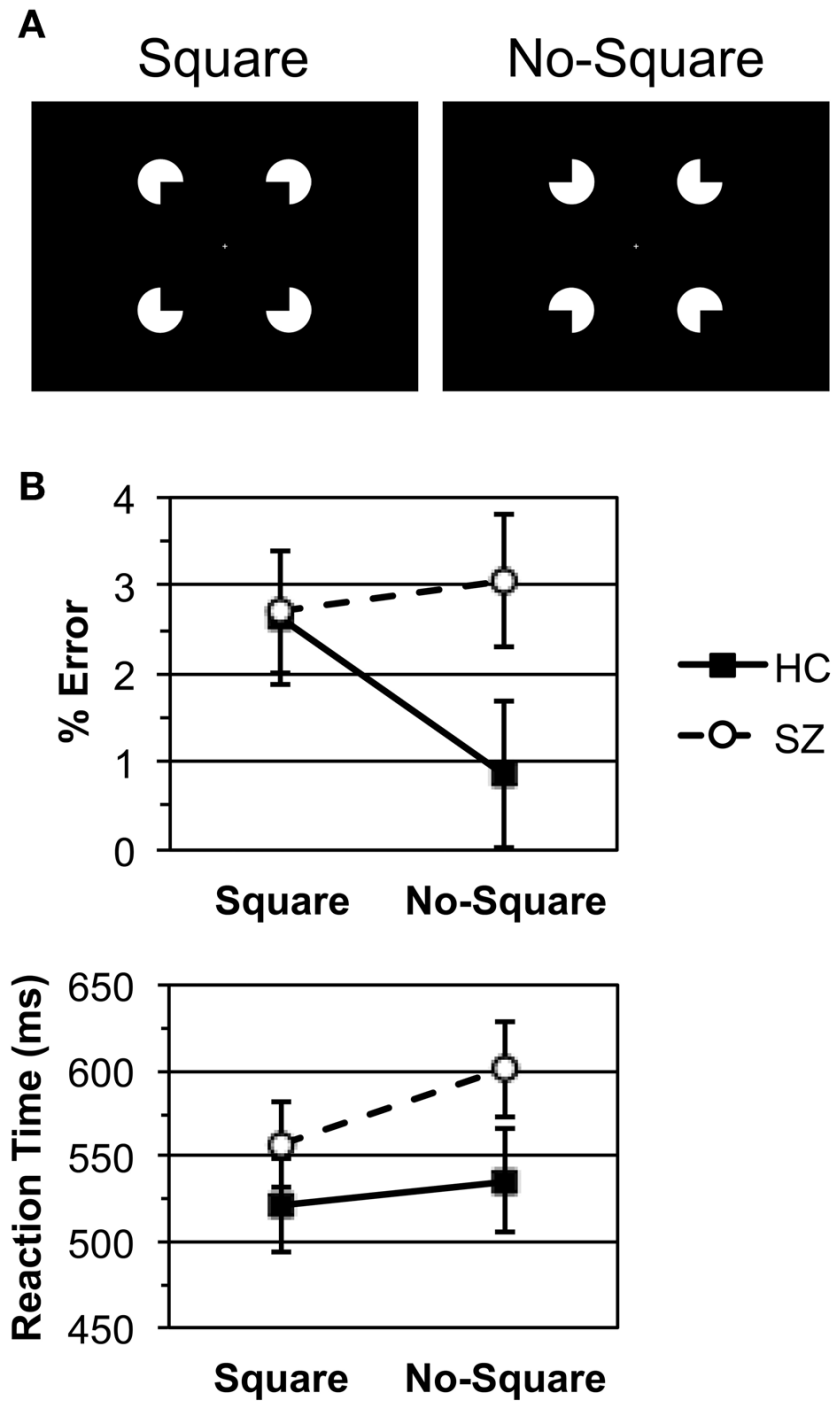
**(A)** The Kanisza Square and No-Square stimuli. **(B)** Error rate and median reaction time data for the healthy control (HC) and schizophrenia patient (SZ) groups. Bars indicate standard error.

The Square and No-Square stimuli were presented in white on a black background at fixation. A fixation cross was continuously present. The stimuli were 5° wide with a 0.4 support ratio (Figure 1A). Unlike our previous studies utilizing Kanisza displays in which the stimuli were presented until after the response had been made (Spencer et al., 2003, 2004), here the stimuli were presented for 106 ms.

### EEG recording and processing

The EEG was recorded with a Biosemi ActiveTwo system using active electrodes in an electrode cap at 71 standard scalp sites (DC–100 Hz bandpass filter, 512 Hz digitization rate). The DC offsets were kept below 25 mV. During data acquisition, all channels were referred to the system's internal loop (CMS/DRL sensors located in the parietal region), and were then re-referenced offline to the left mastoid electrode. The bipolar vertical electro-oculogram (EOG) was derived from electrode Fp1 and an electrode below the left eye. The horizontal EOG was derived from electrodes on the left and right outer canthi.

The epoching and initial processing of the continuous EEG recording were performed with BrainVision Analyzer 2.0 (Brain Products GmbH). For each trial, a 2 s epoch was extracted from 400 ms pre-stimulus to 1598 ms post-stimulus. Further processing was performed using software in MATLAB (Mathworks, Inc.) and IDL (Exelis Visual Information Solutions, Inc.). Error trials were excluded from processing, and an initial artifact detection scan was run. The artifact exclusion criteria were: (1) > ± 90 μV change in one time point; and (2) amplitude range within an epoch exceeding 200 μV. Then independent component analysis [implemented in the *runica.m* program from EEGLAB (Delorme and Makeig, 2004)] was used to remove ocular and muscle artifacts. Independent components representing artifacts were identified based on their characteristic topographic, temporal, and spectral signatures (Keren et al., 2010; Shackman et al., 2010; Hipp and Siegel, 2013). Next, a second artifact detection scan was run. Finally, the retained correct-response, artifact-free epochs were re-referenced to the average reference (Dien, 1998), computed on all 68 scalp channels, excluding the EOG channels. The number of epochs retained per subject was (mean ± standard deviation) 158 ± 13 for HC and 157 ± 17 for SZ, and these numbers did not differ [*t*_(29)_ = 0.300, *p* = 0.766].

Two data sets were created from the single trial epochs: stimulus-locked and response-locked. For the response-locked data set, the original stimulus-locked epochs were shifted according to the reaction time (RT) on each trial such that they encompassed the period from −1448 to 500 ms relative to RT.

Event-related brain potentials (ERPs) and spectral measures were computed from the single-trial epochs. Time-frequency (TF) decomposition was performed using the Morlet wavelet transform (Torrence and Compo, 1998), which was applied in 1 Hz steps from 7–100 Hz at each time point to yield TF maps. The wavelet frequency/duration ratio (f0/σ f) was 6. Here we report results for the phase-locking factor (PLF) measure (Tallon-Baudry et al., 1996). PLF reflects the degree to which EEG phase is consistent across trials, and thus reflects event-locked synchronized activity. This measure is therefore highly redundant with the evoked power measure and tends to be more sensitive to effects on oscillations than evoked power. PLF is computed as one minus the circular variance of phases across trials and ranges from 0 (random distribution) to 1 (perfect phase locking).

Baseline activity was subtracted from each TF map. Baseline periods were from −150 to 0 ms relative to stimulus onset for the stimulus-locked maps, and 0 to 150 ms relative to reaction time (RT) for the response-locked maps.

### Statistical analyses

Subjects' task performance was measured with error rate, median RT, and the signal detection measures *d*′ (discriminability) and log β (response bias) (Wickens, 2002). Effects on ERPs and oscillations were measured with average amplitude/PLF at electrodes and latency windows determined from the grand averages. Performance, ERP, and oscillation measures were analyzed with ANOVAs with the design Group (HC/SZ) X Stimulus (Square/No-Square) (X relevant electrode site factors). The Greenhouse-Geisser correction for inhomogeneity of variance (Keselman and Rogan, 1980) was applied for factors with more than two levels and is reflected in the reported *p-*values.

Correlations between task performance and electrophysiological effects of interest (averaged across electrode sites), demographic, and clinical variables (including symptom ratings) were calculated using the nonparametric Spearman's ρ (2-tailed, α = 0.05).

A statistical non-parametric mapping (SnPM) method based on the permutation test was used to find clusters of TF elements (time points in each frequency band) that reflected Group (HC/SZ) X Stimulus (Square/No-Square) interactions in the response-locked oscillations. The permutation test has several advantages over parametric statistical tests (Nichols and Holmes, 2001; Maris and Oostenveld, 2007). Most importantly for the analysis of TF data, the permutation test does not rely upon assumptions about the distribution of the data. Thus, it is more sensitive than parametric tests when the assumptions underlying the latter are not met (such as normality), which is likely for the PLF measure. Additionally, the permutation test provides control for multiple comparisons, since all the TF elements are permuted in parallel. In practice, however, we found it necessary to apply additional statistical criteria to control for multiple comparisons. Our statistical TF mapping method consisted of the following steps:

1. TF *t*-maps were computed by performing *t*-tests on each TF element across the epoch for each channel. The Group X Stimulus interaction map was computed with between-groups *t*-tests on the Square minus No-Square difference maps, which is mathematically equivalent to a 2 × 2 ANOVA design.
2. Rather than find the corresponding *p*-values of these maps by referring to the *t* distribution, TF maps of the *p*-values were computed for each *t*-map using the permutation test (α = 0.05, 2-tailed, 1000 permutations). To calculate the Group X Stimulus interaction, a difference map (Square minus No-Square) was computed for each subject, and then the assignment of subjects to each group was shuffled on each permutation. The *p*-value of each TF element was obtained by determining the percentile rank of the observed *t*-value in the shuffled *t* distribution. For example, if the observed *t-value* of 3.27 of a TF element had a percentile rank of 90% in the *t* distribution obtained by permutation, the *p*-value of that TF element would be 0.90.
3. The resulting *p*-maps were thresholded at *p* > 0.975 for positive effects (HC > SZ), and *p* < 0.025 for negative effects (SZ > HC). TF elements with *p*-values above/below these thresholds were retained only if they were part of a cluster with a duration of at least 1 cycle of the respective frequency (e.g., 25 ms for a 40 Hz cluster).
4. The thresholded *p*-maps were summed across all 68 scalp EEG channels to create a channel sum histogram of TF clusters. This histogram represents the number of channels on which each TF cluster was found. Each channel sum histogram was thresholded at the 95% percentile of the distribution for that histogram. In other words, if 95% of the TF clusters were present at N channels (channel sums of ≤N), then a TF cluster was considered significant only if it was present on N + 1 channels. The reasoning behind this step was that “true” effects should be present on a multiple channels due to volume conduction.
5. A 1 cycle duration cutoff was applied again to the channel sum histogram, so that all the final TF clusters had to be at least 1 cycle in duration at their respective frequencies. The electrodes contributing to each cluster were then plotted in topographic maps, with color codes indicating the percentage of the cluster area to which the electrode contributed.

## Results

### Task performance

Error rate and RT data are displayed in Figure 1B. HC and SZ were in general highly accurate, with error rates of less than 3% per condition, and did not differ in overall error rate [*F*_(1, 29)_ = 1.31, *p* = 0.261]. HC made more errors for Square than No-Square stimuli, while SZ error rates did not differ between conditions [Group X Stimulus: *F*_(1, 29)_ = 6.20, *p* < 0.05; HC: *F*_(1, 13)_ = 8.02, *p* < 0.05; SZ: *F*_(1, 16)_ = 0.369, *p* = 0.552].

HC and SZ did not differ in overall RT [*F*_(1, 29)_ = 1.67, *p* = 0.207]. Both subject groups responded more quickly to Square than No-Square stimuli [Stimulus main effects: overall *F*_(1, 29)_ = 19.4, *p* < 0.001; HC: *F*_(1, 13)_ = 4.74, *p* < 0.05; SZ: *F*_(1, 16)_ = 16.7, *p* < 0.001]. This effect was larger for SZ than HC [Group X Stimulus: *F*_(1, 29)_ = 4.44, *p* < 0.05].

Analyses of *d*' indicated that both subject groups were able to easily discriminate between the Square and No-Square stimuli HC (mean ± s.e.m., 4.27 ± 0.16) and SZ (4.06 ± 0.22), with no difference between groups [*t*_(29)_ = 0.719, *p* = 0.478]. Log β analyses revealed that HC (0.595 ± 0.176) were biased to make No-Square rather than Square responses [*t*-test vs. 0, *t*_(13)_ = 3.38, *p* < 0.01], whereas SZ (−0.007 ± 0.114) were not biased to make either response. The difference in log β between groups was significant [*t*_(29)_ = 2.97, *p* < 0.01].

The task performance data indicate that the HC and SZ groups performed the task equally well overall, and gave the Square stimuli priority in responding over the No-Square stimuli. However, HC made more misclassifications of Square stimuli. HC did not evince a speed/accuracy tradeoff, as there was no correlation between their RT and error rate Square minus No-Square effects (ρ = 0.433, *p* = 0.122).

### Stimulus-locked ERPs

**Figure 2:**
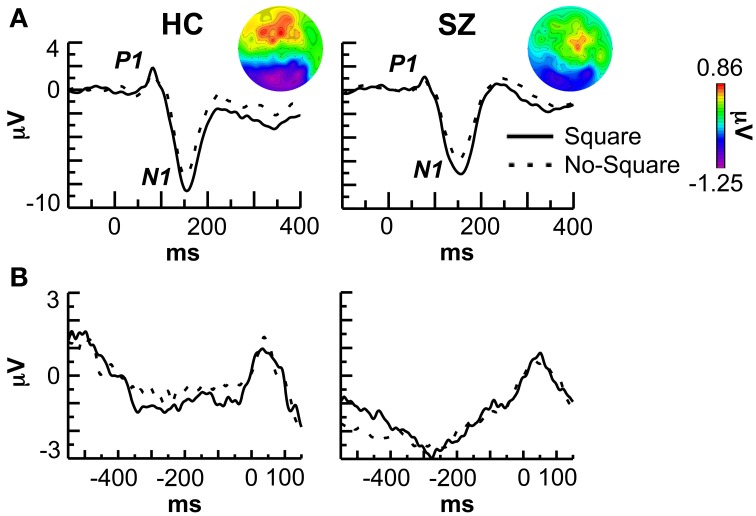
**Event-related potential (ERP) data. (A)** Stimulus-locked ERPs at the P08 electrode. The topography of the negative shift (Square minus No-Square) in each group is shown in the insets. **(B)** Response-locked ERPs at the P08 electrode.

The grand average stimulus-locked ERPs are shown in Figure 2A. The average amplitude of the P1 component was measured at the electrodes PO9/10, PO7/8, PO3/4, and O1/2 in the 70–95 ms latency range. P1 amplitude did not differ between stimulus conditions [*F*_(1, 29)_ = 0.027, *p* = 0.871] but was reduced in SZ compared to HC [*F*_(1, 29)_ = 5.41, *p* < 0.05]. This P1 deficit varied across electrodes [Group X Electrode: *F*_(3, 87)_ = 3.53, *p* < 0.05], being significant at the electrode pairs PO3/4 [*F*_(1, 29)_ = 6.60, *p* < 0.05] and O1/2 [*F*_(1, 29)_ = 7.021, *p* < 0.05].

N1 component average amplitude was measured at the same electrodes as the P1 in the 110–200 ms latency range. N1 amplitude was greater for Square vs. No-Square stimuli overall [*F*_(1, 29)_ = 39.2, *p* < 0.000001] and in each group [HC: *F*_(1, 13)_ = 18.1, *p* < 0.001; SZ: *F*_(1, 16)_ = 21.1, *p* < 0.001] but did not differ between groups [*F*_(1, 29)_ = 0.135, *p* = 0.716]. Further examination revealed that the effect of stimulus on the N1 was actually due to an overlapping long-lasting negative shift in the 110–400 ms range at occipito-temporal electrodes [overall Stimulus: *F*_(1, 29)_ = 19.5, *p* < 0.001] that did not differ significantly between groups [Group X Stimulus: *F*_(1, 29)_ = 1.57, *p* = 0.220; HC: *F*_(1, 13)_ = 11.2, *p* < 0.01; SZ: *F*_(1, 16)_ = 7.36, *p* < 0.05]. The topography of this shift is displayed in the topographic maps in Figure 2A.

### Response-locked ERPs

A sample of the grand average response-locked ERPs are shown in Figure 2B. No effects of stimulus type or subject group were apparent at any electrode.

### Stimulus-locked oscillations

**Figure 3:**
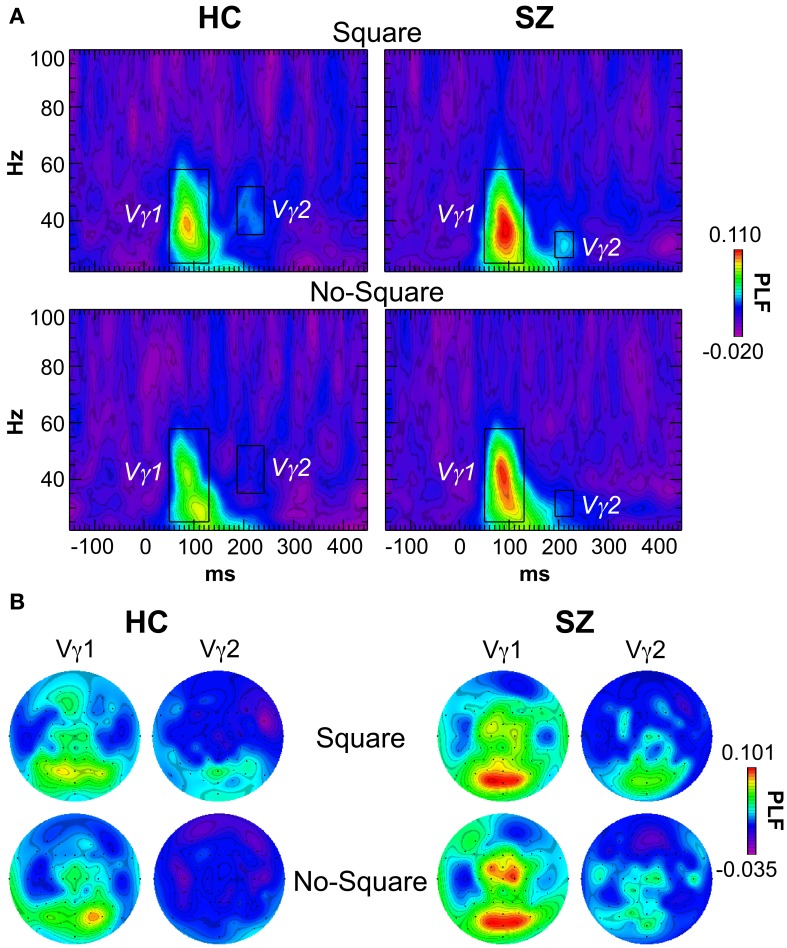
**Stimulus-locked time-frequency (TF) maps of phase-locking factor (PLF). (A)** TF maps averaged across the posterior electrodes in the Square and No-Square conditions for each group. The TF windows within which the Vγ1 and Vγ2 oscillations were measured are indicated with boxes (Vγ1: 50–130 ms, 25–58 Hz; Vγ1 HC: 186–240 ms, 35–52 Hz; Vγ2 SZ: 192–230 ms, 27–36 Hz). **(B)** Topographic maps of PLF for Vγ1 and Vγ2 in the defined TF windows for each condition and group.

The grand average stimulus-locked TF maps averaged across the posterior channels (parietal, occipital, and occipito-temporal electrodes) are shown in Figure 3A. Vγ1 was evoked by Square and No-Square stimuli in both subject groups, with non-significantly higher PLF in SZ than HC. As has been reported previously (e.g., Tallon-Baudry et al., 1997; Spencer et al., 2008a), Vγ1 displayed a bimodal topography (Figure 3B), with components at posterior and fronto-central electrode sites. Therefore, we analyzed the posterior and anterior components of Vγ1 separately.

The average PLF of the posterior Vγ1 component was measured at electrodes P1/2, P3/4, P5/6, P7/8, PO3/4, PO7/8, and O1/2 in the range of 50–130 ms and 25–58 Hz. There were no effects of Group or Stimulus, nor a Group X Stimulus interaction (*F*'s < 0.732, *p*'s > 0.399).

The average PLF of the anterior component of Vγ1 was measured at electrodes F1/z/2, FC3/1/z/2/4, C3/1/z/4, and CP1/z/2 in the same time and frequency ranges as the posterior Vγ1. There were no significant effects of Group, Stimulus, nor a Group X Stimulus interaction (*F*'s < 2.56, *p*'s > 0.121).

A second γ oscillation was observed following Vγ1 at posterior sites, which we termed “Vγ2.” In HC, Vγ2 was evoked by Square stimuli in the range of 186–240 ms and 35–52 Hz. In SZ this oscillation appeared to occur in the nearly the same latency range (192–230 ms) but at a lower frequency (27–36 Hz) in the Square condition. The latency of this oscillation, and the similar topography to Vγ1, suggests that it could be an offset response. Measured in the above latency and frequency ranges at electrodes PO3/z/4, O1/z/2, and Iz, there was a trend for Vγ2 PLF to be increased in the Square relative to the No-Square condition over all subjects [*F*_(1, 29_) = 3.95, *p* = 0.056]. The Group main effect and Group X Stimulus interaction were not significant (*F's < 0.918*, *p's > 0.346*). Since the Stimulus effect on Vγ2 in the SZ group might have been reduced by a small degree overlap with the trailing edge of Vγ1 in the No-Square condition, we analyzed Vγ2 separately in HC, for which there was no overlap with other oscillations. In HC, Vγ2 PLF was significantly increased for Square compared to No-Square stimuli [*F*_(1, 13)_ = 5.27, *p* < 0.05].

### Response-locked oscillations

**Figure 4:**
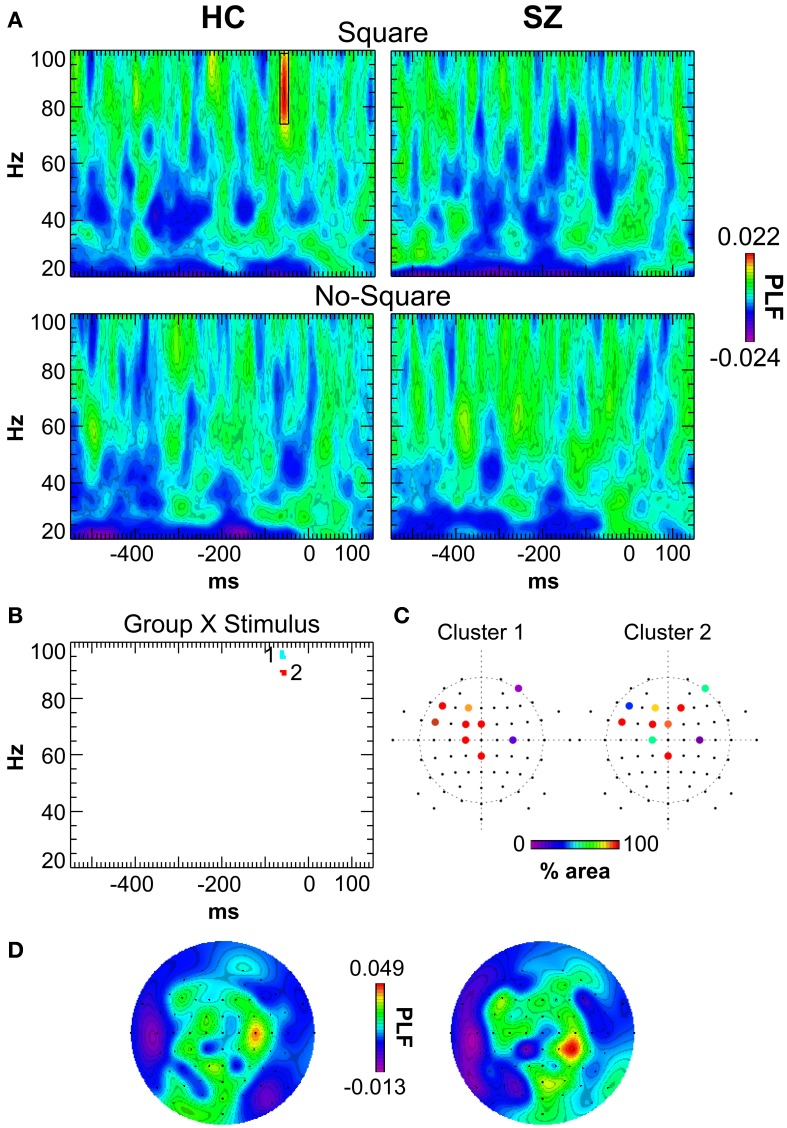
**Response-locked TF maps of PLF. (A)** TF maps averaged across all scalp electrodes for the Square and No-Square conditions for each group. The response-locked γ oscillation in the HC/Square condition is outlined (−69 to −50 ms, 74–99 Hz). **(B)** Group X Stimulus statistical map. The clusters of significant TF elements are indicated. **(C)** Electrodes contributing to each cluster. The percent area of each cluster in which the Group X Stimulus interaction was significant at that electrode is color-coded. **(D)** Topography of the HC/Square response-locked γ oscillation (as indicated by the box in panel A). Left: original data. Right: after switching electrodes between homologous sites in each hemisphere according to subjects' response hand assignment (see Results).

The grand average response-locked TF maps, averaged across channels, are displayed in Figure 4A. The major effect that was apparent was a burst of high γ activity at ~69–50 ms before the button press in the 74–99 Hz band in the Square condition for HC but not SZ.

**Figure 5:**
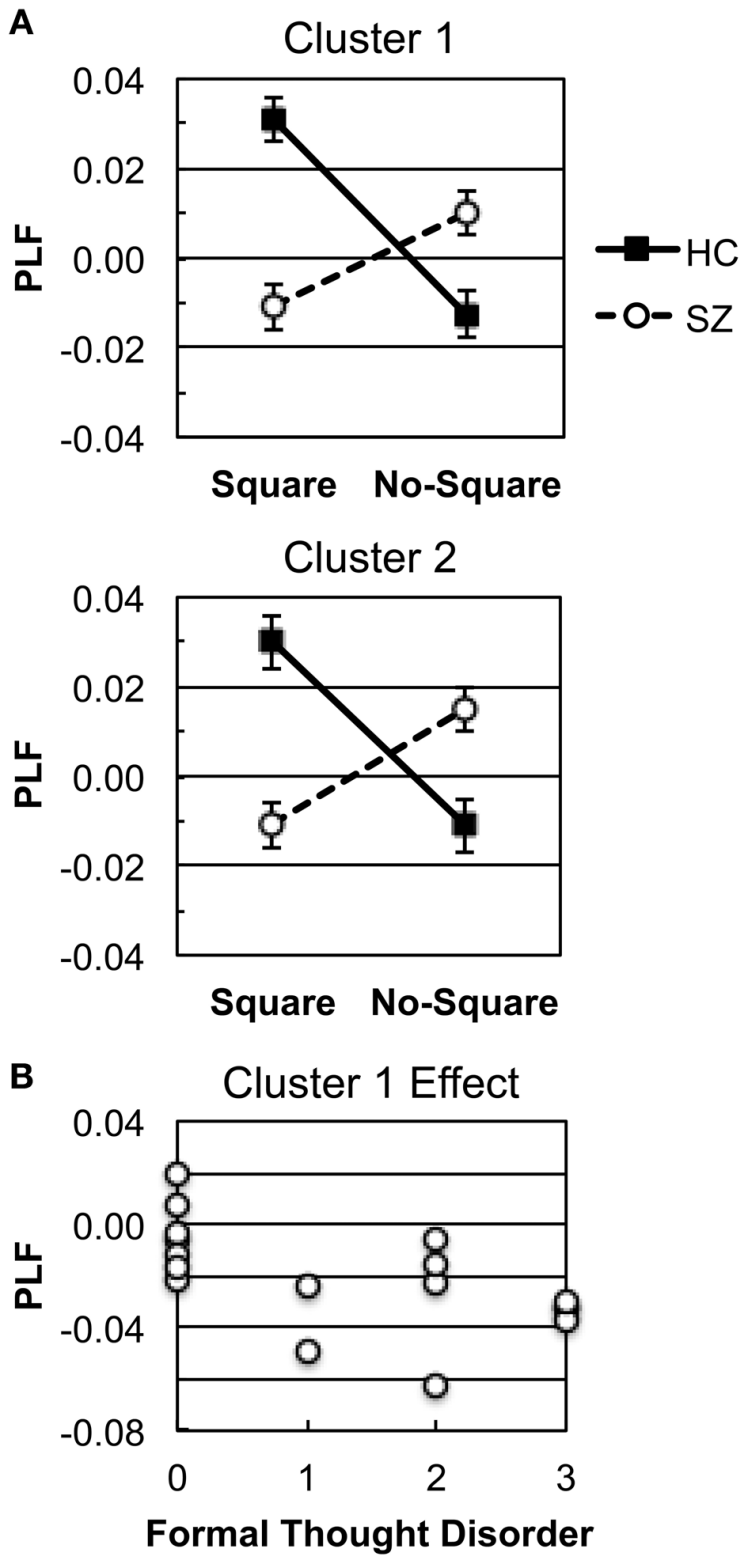
**Response-locked γ oscillation effects. (A)** Square and No-Square condition PLFs in HC and SZ for each Group X Stimulus cluster (see Figure 4B). **(B)** Scatterplot illustrating the correlation between the Cluster 1 effect and the SAPS Global Rating of Formal Thought Disorder scale in SZ.

SnPM was used to detect Group X Stimulus interactions in the response-locked oscillations (see Methods). In the positive (HC > SZ) Group X Stimulus TF map there were 2 clusters of significant TF elements (Figure 4B), both in the high γ band and shortly preceding the response. Cluster 1 spanned the time and frequency ranges of −65.2 to −53.5 ms and 95–97 Hz. Cluster 2 spanned the time and frequency ranges of −65.2 to −51.6 ms and 89–90 Hz. Both clusters had very similar fronto-central topographies (Figure 4C), suggesting that they might reflect the peaks of a broader high γ effect spanning a wider frequency range—namely, the high γ burst apparent in the HC Square data. In follow-up tests for each cluster (Bonferroni-corrected by 2 groups X 2 clusters), HC evinced significant Square > No-Square effects [Cluster 1: *t*_(13)_ = 6.51, *p* < 0.0001; Cluster 2: *t*_(13)_ = 5.92, *p* < 0.001], while SZ demonstrated significant No-Square > Square effects [Cluster 1: *t*_(16)_ = −4.178, *p* < 0.01; Cluster 2: *t*_(13)_ = −4.32, *p* < 0.01] (see Figure 5A). No significant clusters were found in the negative (SZ > HC) Group X Stimulus map.

The predominantly fronto-central topographies of the response-locked γ clusters could be consistent with generators in motor and/or premotor cortex. In fact, decision-related activity has been found in premotor cortex (e.g., Hernández et al., 2002), and response hand-specific accumulation of decision information has been found in γ and β activity in premotor and motor cortex (Donner et al., 2009; de Lange et al., 2013). While the present response-locked γ effects do not seem to reflect pure motor-related activity, as they were modulated by stimulus condition, it is possible that they might be related to some degree to manual response generation. Response hand assignment was counterbalanced across subjects, which could have obscured any lateralization of the cluster topographies associated with particular response hand assignments. That is, approximately half of the subjects pressed the left/right buttons for Square/No-Square, and the other half of subjects had the opposite response hand assignment. Therefore, motor cortex activity in the grand average data should not be lateralized according to response hand.

To investigate the degree to which the response-locked γ effects might be related to motor activity, we tested whether the topography of the response-locked γ oscillation in the grand average data (Figure 4A) would be lateralized according to response hand assignment. We made new grand averages in which the electrodes were switched between the homologous sites in the hemispheres according to the subjects' response hand assignment, so that lateralized activity related to the responses would be made evident. That is, subjects who had the left/No-Square right/Square assignment had their electrodes switched (left hemisphere to right hemisphere and vice versa), so that lateralization of EEG activity related to response hand assignment would be identical across all the subjects. This resulted in only minor changes to the topography of the response-locked γ oscillation, and no increase in its lateralization (Figure 4D).

### Correlation analyses

To test our hypothesis that there would be correlations between response-locked γ effects and positive symptoms (particularly visual hallucinations, thought disorder, and/or disorganization), as in our previous study (Spencer et al., 2004), we investigated whether the response-locked γ Square minus No-Square effects in SZ were correlated with positive symptom rating scales. We focused on the 4 global ratings scales of the SAPS (Hallucinations, Delusions, Bizarre Behavior, and Formal Thought Disorder), and Bonferroni-corrected the correlations for 2 clusters X4 symptom ratings. The Square minus No-Square PLF effects were averaged across the electrodes and TF windows contributing to the Group X Stimulus clusters. These analyses revealed that the Cluster 1 effect was correlated with the Global Rating of Formal Thought Disorder (ρ = −0.676, *p* < 0.05; Figure 5B). Since the direction of the Cluster 1 effect in SZ was for greater PLF in the No-Square than the Square condition, this correlation indicated that SZ with greater thought disorder ratings had larger PLF for No-Square than Square stimuli.

To better understand the functional significance of the EEG effects (Square minus No-Square differences for ERP negative shift, Vγ2 in HC, and response-locked γ clusters), we conducted exploratory correlations between these effects and task performance effects (*d*′, log β, and Square minus No-Square differences in RT and % error). The only correlations were found in HC, for which the RT advantage for Square vs. No-Square stimuli increased with the Vγ2 effect (ρ = −0.554, *p* < 0.05).

None of the significant Square minus No-Square effects in SZ was correlated with medication dosage (ρ's < |0.304|, *p*'s > 0.236).

## Discussion

Gestalt perception was associated with several electrophysiological effects: (1) a negative shift in the ERP that overlapped the N1 component for both HC and SZ; (2) enhanced PLF for Vγ2 in HC (with a possibly similar effect in SZ obscured by overlap with Vγ1); and (3) differences between Square and No-Square conditions in response-locked γ oscillations that differed in direction between HC and SZ. The subject groups' task performance patterns were largely similar. Consistent with our prior study (Spencer et al., 2004), the Gestalt effect on a response-locked γ oscillation was correlated with a positive symptom. Thus, while the exact results of Spencer et al. (2004) were not reproduced, the general pattern of results here is similar in several respects, especially with regard to the sensitivity of response-locked γ oscillations to positive symptoms in SZ.

### Response-locked oscillations

We examined oscillatory activity that preceded and was phase-locked to RT because we reasoned that this approach would reveal oscillations that were more related to the perceptual decision, and hence to the perception of the Gestalt, than to the processing of stimulus features. In our previous study we used a sparse electrode array and analyzed oscillation effects that were apparent via visual inspection. Here we used a dense electrode array with a statistical mapping approach to find Gestalt effects.

In Spencer et al. (2004), illusory Kanisza squares elicited a response-locked γ oscillation in HC and a response-locked β oscillation in SZ. We hypothesized that the β oscillation in SZ was analogous to the γ oscillation in HC, as these oscillations had similar latencies and occipito-temporal topographies, but the oscillation in SZ occurred at a lower frequency. The Gestalt effect on the β oscillation was positively correlated with positive symptom ratings, specifically visual hallucinations, thought disorder, and conceptual organization. In the present study no such β effect was found. Instead, we found response-locked high γ effects that had increased PLF for Square compared to No-Square stimuli in HC, and the opposite sign (No-Square > Square PLF) in SZ. These effects had a fronto-central topography. In SZ, one of these high γ effects was correlated with thought disorder symptoms. Thus, the β oscillation in Spencer et al. (2004) and the high γ oscillation here seem to share a similar sensitivity to positive symptoms, particularly thought disorder.

The high γ response-locked oscillation occurred within 100 ms of the button press and had a mainly fronto-central topography. The latency of this oscillation indicates that it was elicited at a late stage of evidence accumulation, at approximately the same time that the motor response was issued by primary motor cortex (assuming a 50 ms interval between the onsets of electromyographic activity and manual response; Coles et al., 1985). The fronto-central topography of this oscillation is consistent with sources in motor or premotor cortex, where perceptual decision-related activity has been previously reported (e.g., Hernández et al., 2002).

In HC the high γ Gestalt effects consisted of increased PLF for Square compared to No-Square stimuli, while in SZ the effects were in the opposite direction. The increased γ synchronization to a Gestalt stimulus in HC would be consistent with the proposed role for γ oscillations in visual feature binding, but the topography (fronto-central as opposed to posterior) and latency (just preceding the manual response) of the response-locked γ effects do not support such a role. Likewise, the reversed pattern in SZ (No-Square > Square PLF) is not congruent with feature binding. The absence of correlations with task performance measures makes it difficult to posit the functional significance of these effects.

The failure to find the same γ/β effect as in Spencer et al. (2004) could be due to several factors. First, the effect might have been present on too few electrodes and/or was not significant enough to pass the statistical thresholds used in the SnPM procedure. Second, the brief stimulus duration in the present study could have resulted in a different pattern of activity in brain regions involved in evidence accumulation to make the Square/No-Square decision. Third, the use of the average reference here (as opposed to averaged mastoids in the previous study) could have altered the topography of the oscillation.

### ERPs and visual-evoked γ oscillations

Previous studies of illusory contour perception have found a negative ERP shift that overlaps the N1 component for illusory contours compared to control stimuli, which has been localized to the lateral occipital complex (e.g., Murray et al., 2002; Altschuler et al., 2012;. Here this shift was found for HC and SZ and did not differ between them, consistent with the findings of Foxe et al. (2005) and Spencer et al. (2004). In addition, we note that the amplitude of the P1 component was reduced in SZ irrespective of the stimulus, consistent with several prior findings (reviewed in Javitt, 2009).

Several prior studies have reported evidence that visual γ oscillations are generally impaired in schizophrenia [reviewed in Spencer (2008) and Tan et al. (2013)]. In earlier studies our group found that Vγ1 was elicited by illusory Square but not No-Square stimuli in HC, whereas in SZ, neither stimulus elicited this oscillation (Spencer et al., 2003, 2004). We also reported that SZ had reduced Vγ1 PLF in a visual oddball task with letter stimuli (Spencer et al., 2008a). Krishnan et al. (2005) found that the power of the visual steady-state response (VSSR) to β and γ stimulation was reduced in SZ. Likewise, Grützner et al. (2013) reported reductions in SZ of the power of visual-evoked onset and offset γ responses, which are likely to have been analogous to the oscillations we have termed Vγ1 and Vγ2. (The power of a sustained induced γ oscillation was also reduced in SZ.) These findings implied that SZ have a basic deficit in generating γ oscillations to visual stimuli.

However, other evidence suggests that deficits in visual γ oscillations in schizophrenia may not be universal and may depend upon additional factors. In the present study, both types of stimulus in both subject groups evoked Vγ1 as well as Vγ2. Furthermore, Wynn et al. (2005) reported a SZ deficit in γ activity evoked by masked but not unmasked stimuli. In first-episode SZ, Sun et al. (2013) did not find a reduction in the power of a visual-evoked onset γ oscillation, although the power of an offset γ oscillation and sustained induced γ were reduced. Finally, Riečanský et al. (2010) found that the PLF of the 40 Hz VSSR was increased in SZ. Therefore, we must conclude that SZ do not have a basic deficit in generating γ oscillations to visual stimuli, and the visual γ deficits that we have previously reported may be due in part to other problems such as dysfunctional top-down processes. The reason for the difference in Vγ1 results between this study and the earlier ones is not clear. Stimulus duration is unlikely to be a factor, since Spencer et al. (2008a) also utilized a brief stimulus duration (100 ms). At the present we do not have any explanation for the difference in findings, but we will present more data on visual-evoked γ in SZ in future papers.

In the present study we also found a later stimulus-evoked γ oscillation, Vγ2, which had a similar topography as Vγ1, suggesting that it might be an offset response. In HC, Vγ2 PLF was greater in the Square compared to the No-Square condition. This effect was correlated with the difference in RT between stimulus conditions, suggesting that Vγ2 played a functional role in Gestalt perception. Both the Gestalt effect and the correlation with RT were found previously for Vγ1 (Spencer et al., 2003). A similar Gestalt effect might have been present in the SZ group but overlap with the trailing end of Vγ1 prevented us from determining this conclusively. We note that Grützner et al. (2013) reported that Gestalt stimuli (Mooney faces) evoked greater power compared to non-Gestalt stimuli in a visual γ offset response. This Gestalt effect was reduced in the SZ. It is possible that the same effect was observed in both Grützner et al. and the present study (although a reduced Gestalt effect in SZ was not found here).

Vγ2 occurred at the same latency in SZ as in HC but in a lower frequency range. As noted above, a similar difference in the frequency of a response-locked oscillation between HC and SZ was found in Spencer et al. (2004). In that study, Square stimuli elicited a response-locked γ oscillation in HC, whereas in SZ, Square stimuli elicited a response-locked β oscillation. The reason for the difference in frequency between subject groups is not clear.

### Correlations between γ/β oscillations and positive symptoms

In several studies we have reported positive correlations between γ/β measures and positive symptoms of schizophrenia: (1) the β response-locked oscillation Gestalt effect in Spencer et al. (2004), (2) the 40 Hz harmonic of the 20 Hz ASSR (Spencer et al., 2008b), and (3) a left auditory cortex 40 Hz ASSR source (Spencer et al., 2009). While the particular oscillations showing these correlations differ, the general pattern we have found is that increased γ/β is associated with psychosis, even when SZ show a deficit in the oscillation measure compared to HC at the group level. Hence, increased symptomatology has been paradoxically associated with more “normal” oscillatory activity. One possible explanation for these findings is that high-frequency oscillatory activity is downregulated in schizophrenia as a response to neural circuit dysfunctions such as hyperexcitability (e.g., Hoffman and Cavus, 2002), for example as a result of antipsychotic treatment. The failure of this downregulation of γ/β activity is directly related to the persistence of psychotic symptoms, and manifests as across-patient correlations between psychotic symptoms and γ/β effects.

Here we found that a γ Gestalt effect in SZ was correlated with thought disorder symptom ratings, similar to the β Gestalt effect in Spencer et al. (2004). [In support of this finding, we note that Uhlhaas et al. (2004) found relationships between measures of perceptual integration and thought disorder in schizotypal individuals.] However, the direction of the correlation here was negative rather than positive, as the effect was measured as Square minus No-Square PLF, and the SZ had greater PLF in the No-Square than the Square condition. But since SZ had greater PLF in the No-Square condition, and this activity increased relative to PLF in the Square condition, the SZ did show an increase in γ related to positive symptomatology. However, in absolute terms, the degree of abnormality (No-Square > Square PLF) increased with thought disorder symptomatology, such that the patients with the highest thought disorder ratings had the largest No-Square > Square PLF difference. Thus, this effect differs from the above positive correlation findings, in which the most “normal” activity was associated with the most symptoms.

### Conclusions

In this study we attempted to replicate previous findings of abnormal oscillatory indices of visual Gestalt perception in individuals with schizophrenia. While the previous findings were not exactly replicated, in particular the findings of visual-evoked γ deficits in SZ, several aspects were found again. Most notably, a response-locked high-frequency Gestalt effect was found that was correlated with thought disorder symptoms. This finding supports the hypothesis that high-frequency oscillations are sensitive to aspects of psychosis.

### Conflict of interest statement

In the past 3 years, Kevin M. Spencer received consultation fees from Galenea Inc. and Bristol-Myers Squibb. Kevin M. Spencer also received a Research and Development Grant from Galenea Inc. that was administered through the Boston VA Research Institute. The other author declares that the research was conducted in the absence of any commercial or financial relationships that could be construed as a potential conflict of interest.
